# Anatomy of the Long Thoracic Nerve in Relation to Thoracotomy for Spinal Approaches: How to Avoid Nerve Injury?

**DOI:** 10.1227/ons.0000000000001800

**Published:** 2025-10-01

**Authors:** Anhelina Khadanovich, Michal Benes, Radek Kaiser, Jeremy Reynolds, Gerard Mawhinney, Jan Stulik, David Kachlik

**Affiliations:** * Department of Anatomy, Second Faculty of Medicine, Charles University, Prague, Czech Republic;; ‡ Center for Endoscopic, Surgical and Clinical Anatomy (CESKA), Second Faculty of Medicine, Charles University, Prague, Czech Republic;; § 1st Department of Orthopaedics, First Faculty of Medicine, Charles University and Motol University Hospital, Prague, Czech Republic;; ‖ Department of Spinal Surgery, Oxford University Hospitals NHS Foundation Trust, Oxford, UK;; ¶ Nuffield Department of Clinical Neurosciences, University of Oxford, Oxford, UK;; # Department of Spinal Surgery, First Faculty of Medicine, Charles University and University Hospital Motol, Prague, Czech Republic

**Keywords:** Long thoracic nerve, Thoracic disc herniation, Thoracoscopy, Thoracotomy, Winging scapula

## Abstract

**BACKGROUND AND OBJECTIVES:**

Thoracotomy and thoracoscopy are commonly used to access the thoracic spine in cases of vertebral fractures, tumors, spinal deformities, or symptomatic thoracic disc herniations. These procedures involve incisions in the intercostal spaces, which may place the lateral thoracic nerve (LTN) at risk. The LTN is a motor nerve that innervates the serratus anterior muscle, and its injury can result in winging of the scapula. The objective of this study was to describe the anatomic course of the LTN in relation to thoracotomy and thoracoscopic approaches to the thoracic spine.

**METHODS:**

Ten cadaveric specimens embalmed in formaldehyde were dissected bilaterally. Initial dissection was performed in the supine position, followed by repositioning into lateral decubitus. The LTN's location was measured relative to the lateral border of the scapula and the anterior aspect of the spine from the second to seventh intercostal spaces.

**RESULTS:**

The LTN exhibited 3 to 5 branches (mean 3.8 ± 1.0), most frequently at the fourth intercostal space. At the level of the third rib, the nerve's average width was 2.9 ± 0.9 mm. The LTN was located 15.5 to 20.4 mm ventral to the lateral scapular border at the fifth to seventh intercostal spaces and 7.8 to 13.1 mm at more cranial levels. The distance between the nerve and the anterior spinal aspect ranged from 9.9 to 24 mm.

**CONCLUSION:**

To minimize LTN injury, thoracotomy incisions should be placed approximately 15 mm from the scapular border at the second and third intercostal spaces and 35 mm at the fourth to seventh spaces. For thoracoscopy, portal incisions should be made more ventrally, aligned with the anterior spinal border to reduce nerve injury risk.

## Graphical abstract

**Figure:**
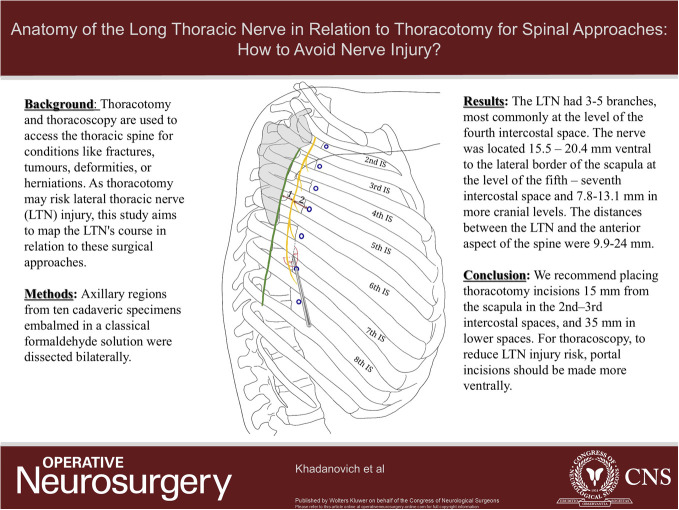

**ABBREVIATIONS:**
IS: intercostal space
LTN: long thoracic nerve.

Thoracotomy is used to approach the thoracic spine in cases of symptomatic thoracic or thoracolumbar disc herniations,^1^ spinal deformities,^2^ tumors,^3^ or fractures of the vertebrae.^4^ Thoracotomy is performed directly above the rib of the involved level in the upper thoracic spine or 2 ribs above the approached vertebra in the lower thoracic spine with the patient in lateral decubitus position.^5,6^ Rib resection may be performed as its fragments may serve as an autograft.^1^ The position of the incision in the intercostal space was variably described in the literature: high thoracic exposure is performed between the spine and scapula, while in other levels, it was described as an unspecified curvilinear incision of the interspace which, according to image documentation, crossed the course of the long thoracic nerve.^3,6-9^ In vertebral and paravertebral tumors, the length and position of the incision varies according to the extent of the tumor.^3,10^ Thoracoscopy, which gained in popularity over the past 2 decades, has a wide spectrum of indications, including spinal biopsy procedures, debridement, discectomy, decompressions, corpectomies, interbody fusions, and internal fixations.^11^ Moreover, incisions for thoracoscopy portals are also associated with the possibility of long thoracic nerve injury with consequent development of a winged scapula. The winging scapula was described in 3.3% to 5% of patients undergoing posterolateral thoracotomy.^12-14^ The most common etiology of the winging scapula is direct trauma to the long thoracic nerve, which causes palsy of the serratus anterior muscle. Consequently, the patient is unable to do and maintain external rotation and protraction of the scapula and abduction of the shoulder above the horizontal level.^15^ Management of winged scapula typically requires prolonged physical therapy and represents a significant source of discomfort and pain.^15^ Therefore, the aim of our study was to describe the course of the lateral thoracic nerve (LTN) in the context of thoracotomy and thoracoscopic approaches to the thoracic spine.

## METHODS

**FIGURE 1.**
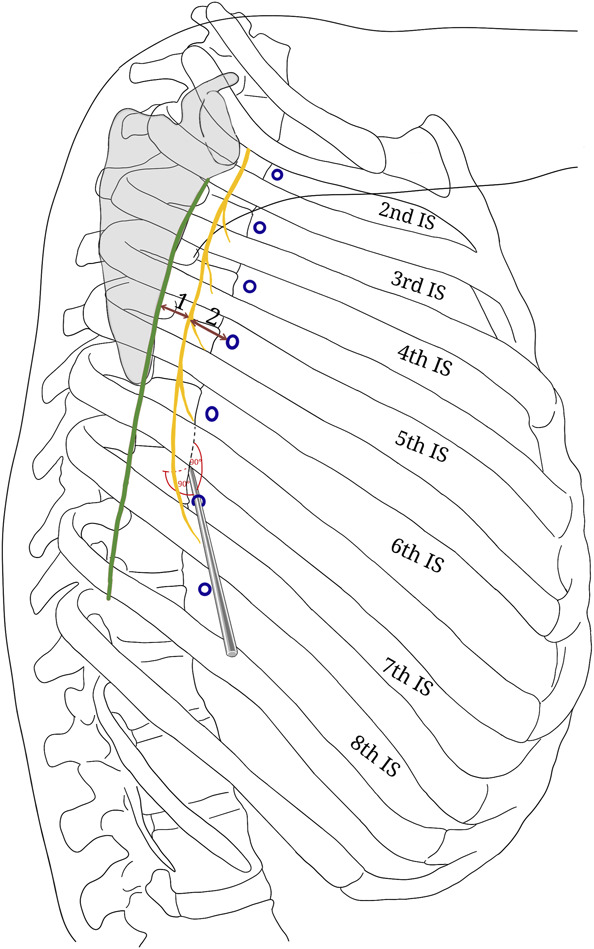
Figure showing measurements. 1—distance between the lateral border of the scapula (green line) and the LTN (yellow); 2—distance from the LTN to the rod piercing the thoracal wall (blue circles) representing the projection of the anterior side of the spine at the wall. IS, intercostal space; LTN, lateral thoracic nerve.

Ten cadaveric specimens (4 men and 6 women; mean age 71.8 years) embalmed in a classical formaldehyde solution were used for the study. All cadavers were obtained from the Informed Donation Program of the Department of Anatomy, Second Faculty of Medicine, Charles University, Prague, Czech Republic. Axillary region was dissected with the specimen laying supine with abducted arm. First, a skin covering the pectoralis major muscle and the axilla to the lateral margin of the latissimus dorsi muscle was removed. Then, the pectoralis major and pectoralis minor muscles were detached from their origins and reflected laterally. The margin of the latissimus dorsi muscle was identified, and the LTN was carefully dissected superficially to the serratus anterior muscle. The entire course of the LTN and its branches were documented. Next, each specimen was repositioned in the lateral decubitus position with the arm fixed at 90° ventral flexion. Using pins, the lateral margin of the scapula was marked and extended caudally along the course of the LTN. At the levels of the second through seventh intercostal spaces, we measured the distance from the nerve to the lateral scapular border. Then, at each of these interspaces, a small incision was made along the anterior axillary line and a long, thin metal rod was inserted perpendicular to the dissection table until it reached the anterior aspect of the corresponding vertebral body (Figure 1). The distance from the LTN to this rod—which represented the anterior edge of the vertebral body—was recorded at each level.

The study protocol was approved by the local institutional ethical commission (No. EK-22/25), and procedures were performed in accordance with The Code of Ethics of the World Medical Association (Declaration of Helsinki) and its later amendments.

For all measurements, a digital caliper (SometCz) with an accuracy of 0.03 mm was used. Photographs were provided using Kodak Astro Zoom AZ252.

### Statistical Analysis

Continuous variables are presented as mean values with SD. Categorical variables are presented as absolute values with frequencies. The Mann–Whitney *U* test was used for comparison of side differences with a *P* value ≤ .05 indicating statistical significance. The statistical analysis was performed in GraphPad Prism v. 9.5.1 (GraphPad Software).

## RESULTS

**FIGURE 2.**
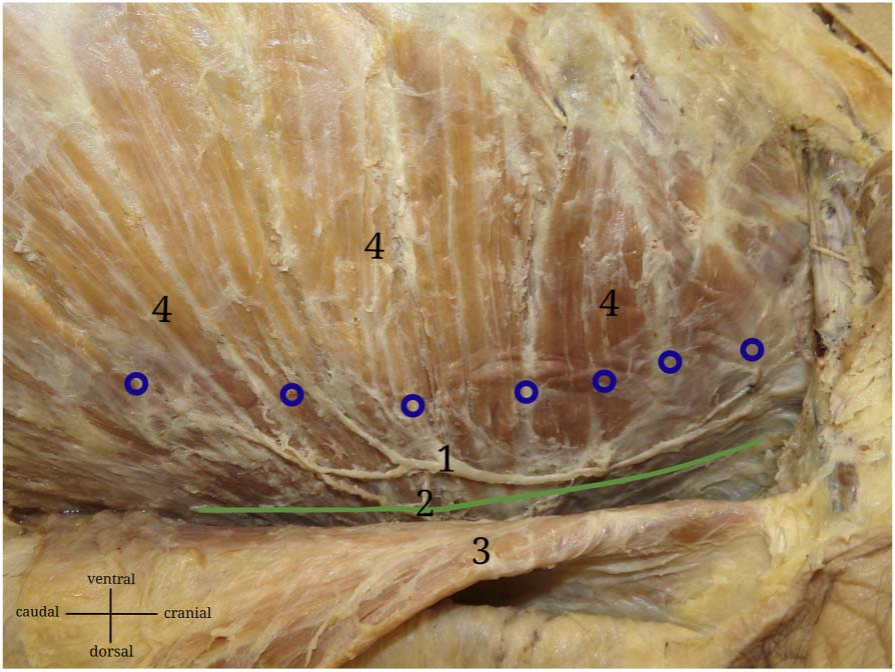
Photography showing the left (1) crossing the lateral thoracic artery (2) on the serratus anterior muscle (4). The latissimus dorsal muscle (3) was reflected laterally. The green line represents the lateral border of the scapula in lateral decubital position. Blue circles showing the projection of the anterior side of the spine on the thoracic wall.

The LTN terminated at the level of the sixth rib in 10 (50%) of cases, seventh rib in 6 (30%) cases, and eighth rib in 4 (20%) cases. At the level of the third rib, the average width of the nerve was 2.9 ± 0.9 mm. The LTN gave off between 3 and 5 branches (mean 3.8 ± 1.0), most commonly at the level of the fourth intercostal space (IS) (Table 1; Figure 2). In the lateral decubitus position, the inferior angle of the scapula was located at the level of the fifth IS (Table 1).

**TABLE 1.** Table Summarizing the Levels Where the Scapular Tip, the LTN Termination, and the LTN Branching Occurred

Level	Scapular tip	LTN termination	LTN branches
3rd rib	0	0	8 (11%)
3rd intercostal space	0	0	8 (11%)
4th rib	0	0	10 (13%)
4th intercostal space	0	0	13 (17%)
5th rib	4 (20%)	0	9 (12%)
5th intercostal space	12 (60%)	0	10 (13%)
6th rib	2 (10%)	10 (50%)	5 (6%)
6th intercostal space	2 (10%)	0	5 (6%)
7th rib	0	6 (30%)	5 (6%)
7th intercostal space	0	0	3 (4%)
8th rib	0	4 (20%)	0

LTN, lateral thoracic nerve.

Measurements of the distances between the LTN and both the lateral edge of the scapula and the anterior surface of the vertebral body at the levels of the second to seventh intercostal spaces are summarized in Table 2.

**TABLE 2.** Table Demonstrating Distances From the LTN to the Lateral Margin of the Scapula and to the Anterior Side of the Vertebral Body in the Second-Seventh IS

Parameter	Total	Left	Right	*P* value
LTN—scapular edge 2nd IS	7.8 ± 8.3 (4.2 to 11.4)	7.6 ± 5.5 (4.2 to 11.0)	8.0 ± 10.7 (1.4 to 14.6)	.531
LTN—scapular edge 3rd IS	9.0 ± 6.3 (6.2 to 11.8)	9.7 ± 5.4 (6.4 to 13.0)	8.3 ± 7.2 (3.8 to 12.8)	.888
LTN—scapular edge 4th IS	13.1 ± 8.7 (9.3 to 16.9)	14.6 ± 6.2 (10.8 to 18.4)	11.2 ± 10.8 (4.5 to 17.9)	.725
LTN—scapular edge 5th IS	15.5 ± 13.0 (9.8 to 21.2)	17.3 ± 11.5 (10.2 to 24.4)	13.6 ± 14.6 (4.6 to 22.6)	.190
LTN—scapular edge 6th IS	17.5 ± 13.8 (11.5 to 23.5)	18.1 ± 13.9 (9.5 to 26.7)	16.9 ± 14.5 (7.9 to 25.9)	.755
LTN—scapular edge 7th IS	20.4 ± 16.2 (13.3 to 27.5)	20.7 ± 20.2 (8.2 to 33.2)	20.1 ± 15.3 (10.6 to 29.6)	.830
LTN—ant. vert. body 2nd IS	−14.1 ± 8.9 (−18.0 to −10.2)	−12.0 ± 10.1 (−18.3 to −5.7)	−16.2 ± 7.4 (−20.8 to −11.6)	.432
LTN—ant. vert. body 3rd IS	−17.0 ± 9.1 (−21.0 to −13.0)	−14.9 ± 11.3 (−21.9 to −7.9)	-19.0 ± 6.2 (-22.8 to −15.2)	.714
LTN—ant. vert. body 4th IS	−19.3 ± 11.0 (−24.1 to −14.5)	−16.6 ± 13.2 (−24.8 to −8.4)	−22.0 ± 8.1 (−27.0 to −17.0)	.726
LTN—ant. vert. body 5th IS	−23.3 ± 11.7 (−28.4 to −18.2)	−19.3 ± 13.8 (−27.9 to −10.7)	−27.4 ± 7.8 (−32.2 to −22.6)	.704
LTN—ant. vert. body 6th IS	−24.0 ± 10.5 (−28.6 to −19.4)	−21.4 ± 11.4 (−28.5 to −14.3)	−26.6 ± 9.4 (−32.4 to −20.8)	.895
LTN—ant. vert. body 7th IS	−9.9 ± 13.6 (−15.9 to −3.9)	−8.8 ± 19.1 (−3.0 to −20.6)	−11.7 ± 0.9 (−12.3 to −11.1)	.230

IS, intercostal space; LTN, lateral thoracic nerve.

Measurements are presented in millimeters. Positive numbers indicate the was ventrally to the scapular margin. Negative numbers show that the was dorsally to the anterior side of the vertebral body. 95% CIs are attached in square brackets.

## DISCUSSION

Symptomatic thoracic herniation occurs in around 1 patient in a million cases,^16,17^ it is often accompanied by the compressive myelopathy and debilitating pain which are main indications for surgical intervention.^18^ Moreover, the thoracic region is the most site for spine metastases.^19^ The thoracic spine is preferably approached from the anterior aspect through the transthoracic or thoracoscopic approach, ensuring excellent exposure of the ventral spine, without the need to manipulate the dura, and the ability to preserve stability without fusion.^18^ These procedures are not usually performed on a daily basis by spine surgeons as the learning curve is steep.^20^ For this reason, knowledge of anatomic relationship of surrounding structures is of great importance to enhance the approach with minimal complications.

The LTN is a somatomotor nerve that commonly arises from the C5, C6, and C7 roots.^21-23^ The nerve travels deep to the clavicle, posterior to the axillary artery and terminates superficially on the serratus anterior muscle to which it provides innervation.^21^ According to Wang et al,^22^ the LTN has 1-4 branches to each of superior 6 slips of the serratus anterior muscle, while the first slip is innervated by the superior trunk of the brachial plexus. The nerve branches to the inferior slips penetrate through the sixth muscle slip and continue intramuscularly as a deep portion. Similarly, we found that the superficial portion of the LTN terminated at the level of sixth rib in most of our cases, where the sixth slip of the serratus anterior muscle is found.

The relationship of the LTN to the midaxillary line was previously described by O et al, who observed 17 Korean cadaveric specimens in the supine position.^24^ The authors recommended that midaxillary thoracotomies for cardiothoracic procedures should be performed at a distance of 4 cm anterior to the midaxillary line in the fourth to sixth intercostal spaces to avoid injury to the LTN. However, these data are not directly applicable to posterolateral thoracotomy, which is performed with the patient in the lateral decubitus position, where the arm is fixed in forward flexion and the scapula is displaced. Therefore, the aim of our study was to describe the position of the LTN relative to both the scapula and the spine in the lateral decubitus position along the entire course of the nerve, to provide more anatomically relevant information for thoracic spine approaches. Another study by Salazar et al analyzed the location of the LTN relative to the tip of the scapula and at 2-cm intervals cranially along the lateral scapular margin (up to 6 cm) in specimens positioned in the lateral decubitus position.^25^ Although their body positioning was consistent with ours, we found that the LTN was located significantly closer to the lateral scapular margin at comparable levels (15.5–17.5 mm in our study vs 31 to 45 mm in Salazar et al^25^). Nonetheless, both studies noted a consistent tendency for the nerve to course closer to the scapula at more cranial levels. In addition, we quantified the distance between the LTN and the anterior surface of the vertebral body by inserting a thin rod toward the vertebra in the second to seventh intercostal spaces. These measurements provide valuable information about the nerve's relationship to a fixed anatomic landmark—the vertebral column. By contrast, the scapula is mobile, and its position is influenced by the degree of arm flexion. This mobility may partly explain discrepancies between previously reported measurements^25^ and those observed in our study. Knowledge of the position of the LTN in relation to the spine is crucial in assessing where to place the incision to avoid the LTN injury, especially in thoracoscopic approach. In this technique, the surgeon should visualize the projection of the spine onto the thoracic wall to determine the optimal placement of the portals.

The thoracoscopic approach is associated with insertion of 4 portals through 15-mm long incisions.^26^ According to the position of the portals and our data about the localization of the nerve to the vertebra, we anticipate that the LTN is at risk of injury during the insertion of portals along the spine axis, that is the working portal and the portal for endoscopic camera because they are placed above the vertebra where the LTN is located 9.9 to 24 mm dorsal to the anterior aspect of the spine. Thus, we recommend placing the incisions more ventrally, aligning the anterior border of the spine. If the LTN is encountered during the incision, prolonging the incision in ventral direction is advised to avoid the nerve injury. The thoracoscopic approach is associated with incisions at different levels in comparison with the thoracotomy where the incision is longer and placed in one IS. Incisions in more cranial levels are associated with a higher risk of developing the winging scapula, while branching of the LTN caudal to the scapular tip assures preservation of most of the lower serratus anterior muscle innervation which plays important role in scapula stabilisation.^25^ Even in lesions located in the middle and low thoracic spine, it should be considered that approach to these levels is still associated with a risk of LTN injury because the endoscopic portal is inserted cranially to the working portal in case of thoracoscopy as well as the incision being placed 2 levels above the lesion in thoracotomy.^7^

**FIGURE 3.**
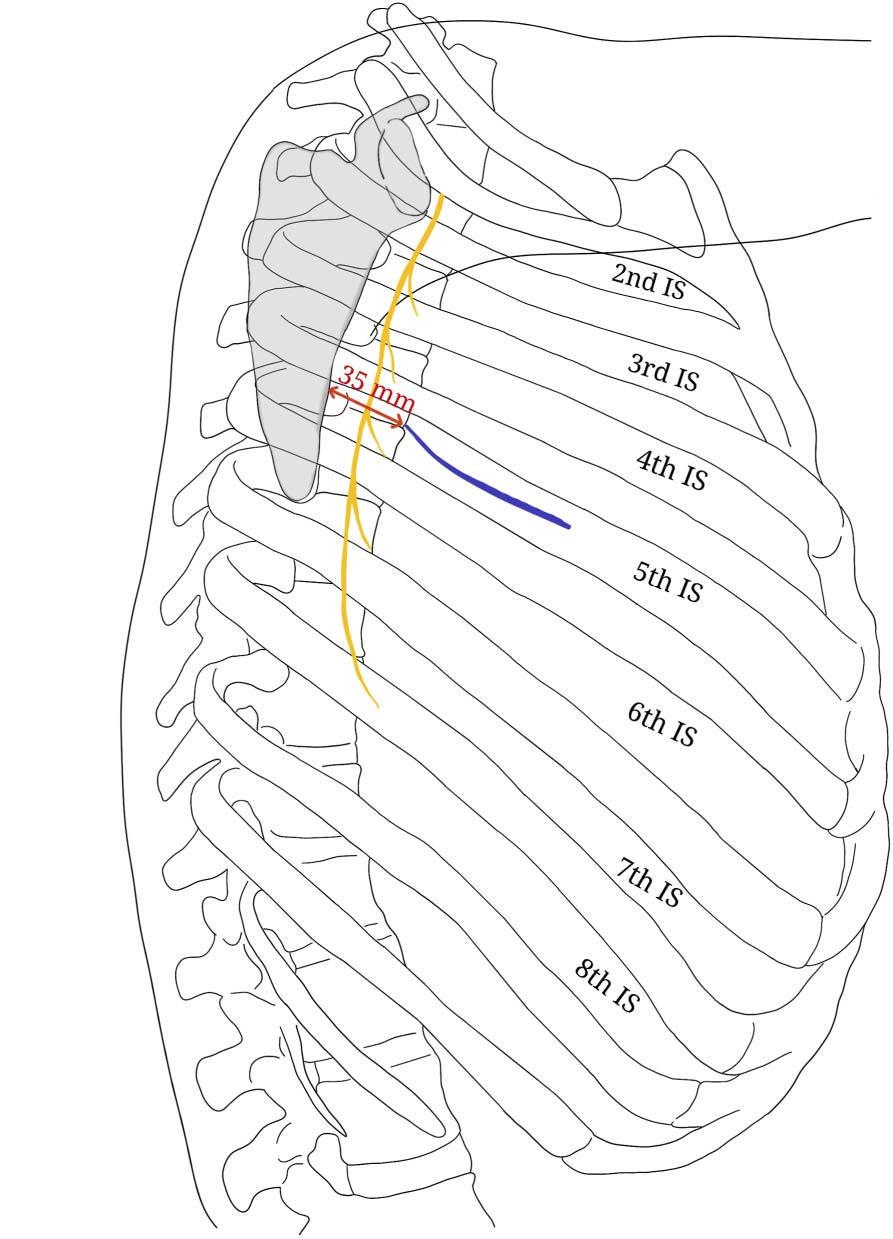
Thoracotomy incision (blue) in the fifth IS (5th IS), positioned 35 mm from the lateral border of the scapula to avoid injury to the (yellow). IS, intercostal space.

The side of the thoracotomy incision is chosen according to the vascular anatomy and also asymmetry of the lesion. The T4-T6 levels are usually approached through right thoracotomy, whereas lesions under the T7 level are approached from the left side.^1^ The assistance of a thoracic surgeon may be required to provide adequate exposure.^1^ In the upper thoracic spine (T3-T4) a stick-shaped, incision is performed along the medial border of the scapula, and under the T4 level, the incision is extended 4 cm from the posterior midline to the posterior axillary line.^5^ Right infra-axillary thoracotomy, which is performed through the incision from the armpit to nipple along the lateral border of the pectoralis major muscle, was described to provide excellent direct view at the spine T2-T6 levels.^9^ However, there is a high risk of injury the LTN in upper part of the serratus anterior muscle. To approach giant paraspinal thoracic schwannomas larger incision is required, starting from the midaxillary line and extending posteriorly around the scapular tip.^3^ To access giant thoracic herniations, the curvilinear incision extends from the posterior angle of the rib to the nipple line.^18^ Some studies also describe the thoracotomy for thoracic spine approach as unspecified incision in interspace.^6,20,27^ According to our data, the LTN is found in 7.8 to 20.4 mm from the lateral scapular border in lateral decubitus position with 90 degrees of ventral flexion in shoulder. The nerve is closer to the lateral border of the scapula in cranial direction, that is why we recommend performing the incision at minimum of 15 mm from the lateral border of the scapula at the second and third IS and minimum of 35 mm from the lateral border of the scapula in the fourth-seventh intercostal spaces (Figure 3).

Winging scapula is frequently caused by LTN injury leading to serratus anterior muscle paralysis. Clinically, patients present with winging of the medial scapula, restricted motion, and pain,^28^ which can improve in time but significant portion of patients cannot continue their manual jobs due to long-term pain and shoulder dysfunction.^29^ As the winging scapula is described rarely as a complication of the thoracotomy or thoracoscopy for thoracic spine approach and these procedures are not performed on a daily basis, the corresponding literature is limited. Safaei et al reported a case of scapular winging after thoracotomy for right thoracic scoliosis with subsequent development of winging scapula with EMG-confirmed LTN injury.^2^ A posterolateral thoracotomy incision is also widely used in children to treat congenital heart diseases with reported rate of 77% of winging scapula after the incision in the fourth IS.^30^ Another case of the winging scapula was described in a patient after thoracotomy for lung cancer performed in the fourth IS.^31^ The authors admit that they had no experience with the serratus anterior muscle paralysis even after posterolateral thoracotomy and attribute it to the low level at which the LTN is cut. In larger number of patients, 5.7% of winging scapula occurred after posterolateral thoracotomy.^14^

### Limitations

Our study was performed on embalmed cadaveric specimens, leading to a possible soft-tissue shrinkage. This fact should be considered when translating our results into clinical practice. Moreover, the number of specimens analyzed in this study was relatively low, which may have contributed to the observed high variability in the results. In addition, the distance to the anterior border of the spine was measured by inserting a metal rod perpendicular to the dissection table, with the specimen positioned in the lateral decubitus position. This method, while practical, may be affected by slight variations in specimen positioning or anatomic deformities of the spine or thoracic wall, which could be a source of systematic error. Moreover, the rod was not inserted under any imaging control. In addition, our measurements were performed in the lateral decubitus position of the specimen with 90 degrees of ventral flexion which influence the position of the scapula. Therefore, our measurements are applicable for patients in the same positioning.

## CONCLUSION

Based on our experimental data, we recommend avoiding thoracotomy incisions up to 15 mm from the lateral border of the scapula in the second and third IS and up to 35 mm from the lateral border in the fourth-seventh intercostal spaces when the patient is positioned in the lateral decubitus. In the thoracoscopic approach, the insertion of the working portal and portal for endoscopic camera pose a risk for the LTN injury; thus, we recommend performing the incision more ventrally, aligning the anterior border of the spine.
